# Disease-Associated Mutations in the *HSPD1* Gene Encoding the Large Subunit of the Mitochondrial HSP60/HSP10 Chaperonin Complex

**DOI:** 10.3389/fmolb.2016.00049

**Published:** 2016-08-31

**Authors:** Peter Bross, Paula Fernandez-Guerra

**Affiliations:** Research Unit for Molecular Medicine, Department of Molecular Medicine, Aarhus University and Aarhus University Hospital Aarhus, Denmark

**Keywords:** HSP60, chaperonin, neurological disease, protein folding problem, mitochondria, gene variation

## Abstract

Heat shock protein 60 (HSP60) forms together with heat shock protein 10 (HSP10) double-barrel chaperonin complexes that are essential for folding to the native state of proteins in the mitochondrial matrix space. Two extremely rare monogenic disorders have been described that are caused by missense mutations in the *HSPD1* gene that encodes the HSP60 subunit of the HSP60/HSP10 chaperonin complex. Investigations of the molecular mechanisms underlying these disorders have revealed that different degrees of reduced HSP60 function produce distinct neurological phenotypes. While mutations with deleterious or strong dominant negative effects are not compatible with life, *HSPD1* gene variations found in the human population impair HSP60 function and depending on the mechanism and degree of HSP60 dys- and mal-function cause different phenotypes. We here summarize the knowledge on the effects of disturbances of the function of the HSP60/HSP10 chaperonin complex by disease-associated mutations.

## Introduction

**Figure 1:**
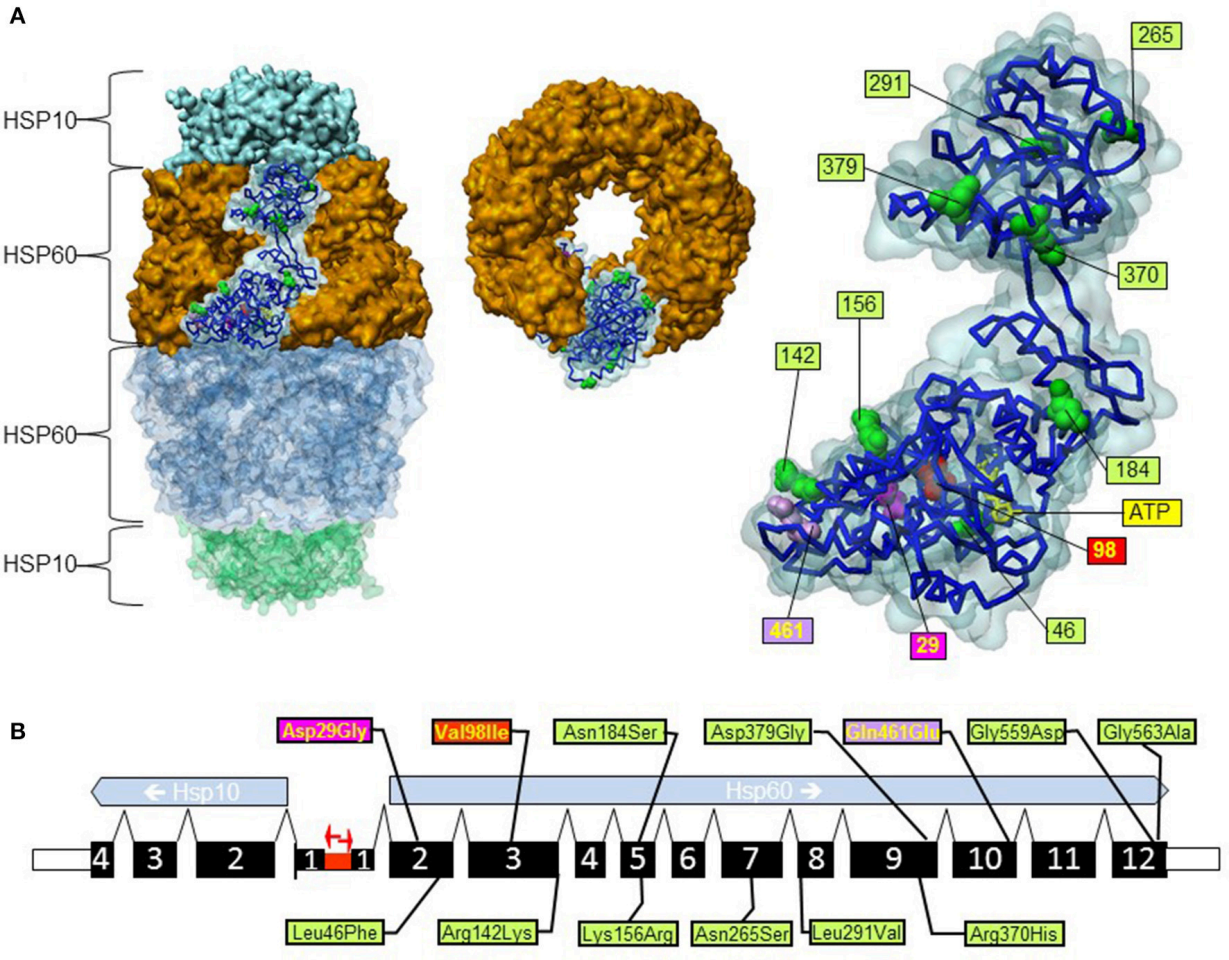
**Position of gene variations in the *HSPD1* gene and the HSP60 protein structure. (A)** Structure of the HSP60/HSP10 complex and position of mutations. 3d structure representations were created based on PDB coordinates 4pj1 (Nisemblat et al., 2015) using the software Discovery Studio 4.5.0.15071 (Biovia). Left: surface representation of the structure of the HSP60/HSP10 complex; HSP60 and HSP10 rings are indicated. The surface representation of one subunit of the upper HSP60 ring is shown transparent and the carbon backbone and space filling representation of amino acid positions where missense variations have been found are shown. Middle: upside view of the upper HSP60 ring. Right: enlarged view of the highlighted subunit from the complex shown on the left with numbering of positions with missense variations. The mutations p.Gly559Asp and p.Gly563Ala are not shown because the C-terminal part of the HSP60 protein is not contained in the crystal structure. The bound ATP molecule is shown as yellow sticks. **(B)** Exon structure of the human *HSPD1 and HSPE1* genes encoding HSP60 and HSP10. The two genes are situated in a head to head configuration on chromosome 2 with a common bidirectional promoter (red arrows). Exons are numbered and their coding parts are given as broad bars. Positions of missense variations are shown; color coding is in relation to disease-association (see text).

The type I chaperonins, a subclass of the molecular chaperone family of proteins, assist folding of proteins in the bacterial cytosol, the mitochondrial matrix space, and the chloroplast stroma. Like its bacterial and chloroplast homologs the mitochondrial HSP60/HSP10 complex is composed of two seven-meric rings of the large subunit (HSP60) stacked back to back (Nisemblat et al., 2015; Figure 1A). The HSP60 ring structures enclose an inner cavity that is sealed by lids formed by seven-meric rings of the small subunit (HSP10). With the exception of a few endosymbionts, homologs of these proteins are abundantly expressed in mitochondria, chloroplasts and bacteria. The functional folding cycle of the mammalian HSP60/HSP10 complex (Nielsen and Cowan, 1998; Levy-Rimler et al., 2001, 2002) has to a large degree been elucidated in analogy to detailed studies of the homologous GroEL/GroES complex of *E. coli* bacteria (Horwich, 2013; Hayer-Hartl et al., 2015). Cycles including binding of proteins undergoing folding to the HSP60 rings, their encapsulation by association of HSP10 rings and dissociation of both the HSP10 ring and the enclosed protein are orchestrated by ATP binding, hydrolysis and release of ADP by the HSP60 subunits. These cycles promote folding of proteins to the native state, but not every cycle results in successful folding. Some proteins may require several rounds. Knock-out experiments have shown that the genes encoding homologs of the HSP60/HSP10 complex are essential in organisms from bacteria to mice (Cheng et al., 1989; Fayet et al., 1989; Perezgasga et al., 1999; Christensen et al., 2010). In humans only few gene variations altering the amino acid sequence of HSP60 and HSP10 have been described (Table 1). However, very rare disease-associated missense mutations in HSP60 have been associated with a dominant form of hereditary spastic paraplegia (HSP; Hansen et al., 2002) and a recessively inherited white matter disorder called MitCHAP60 disease (Magen et al., 2008). In another article published under this research topic we describe the first potentially disease associated mutation in HSP10 that has been identified in a patient with a neurological and developmental disorder (Bie et al., submitted).

**Table 1:** **Missense variations in the *HSPD1* gene encoding HSP60**.

**Variation**	**Disease association**	**Growth in genetic complementation assay**	**ExAC allele count**	**PolyPhen-2 prediction^#^**	**References**
p.Asp29Gly	MitCHAP60	Slow, temperature-sensitive	0	benign	Magen et al., 2008
p.Leu46Phe		Not tested	29	possibly damaging	ExAC
p.Val98Ile	SPG13	No growth	0	possibly damaging	Hansen et al., 2002
p.Arg142Lys		Not tested	56	benign	ExAC
p.Lys156Arg		Not tested	17	benign	ExAC
p.Asn184Ser		Unaffected	61	benign	Hansen et al., 2002
p.Asn265Ser		Not tested	23	possibly damaging	ExAC
p.Leu291Val		Not tested	12	probably damaging	ExAC
p.Arg370His		Not tested	13	benign	ExAC
p.Asp379Gly		Unaffected	0	benign	Bross et al., 2007
p.Gln461Glu	SPG13^*^	Impaired	0	probably damaging	Hansen et al., 2007
p.Gly559Asp		Unaffected	0	possibly damaging	Bross et al., 2007
p.Gly563Ala		Unaffected	1904	probably damaging	Bross et al., 2007

*Missense variations recorded in the literature and/or in the ExAC consortium database with ≥10 alleles are shown. The basis for scoring growth in the genetic complementation assay is explained in Section Functional analysis in vivo. Prediction of possible impact of amino acid substitutions was performed using the PolyPhen-2 webserver (Adzhubei et al., 2010)*.

# *PolyPhen-2 has three prediction output options, from the highest probability score for being damaging to the lowest: “probably damaging,” “possibly damaging” and “benign.” The asterisk denotes that the variation/disease relationship for the p.Gln461Glu variation is not fully established. For further details see text*.

## Amino acid sequence variations in HSP60

The genomic structure for the human *HSPD1* and *HSPE1* genes encoding the proteins HSP60 and HSP10, respectively, has been characterized experimentally (Hansen et al., 2003). The *HSPD1* and *HSPE1* genes are localized at chromosome locus 2q33.1 in a head to head arrangement with a bidirectional common promoter (Figure 1). Such a head to head arrangement is evolutionary conserved from *C. elegans* to humans (Bross, 2015). The two genes comprise ~17 kb and consist of 12 and 4 exons, respectively (Figure 1B). The first exon of Hsp60 is non-coding and the first exon of Hsp10 contains only the first codon (Hansen et al., 2003). At present there are two amino acid variations in HSP60 known for which a clear disease-association has been established: the HSP60-p.Val98Ile mutation associated with a dominantly inherited form of HSP (SPG13; OMIM #605280; Hansen et al., 2002) and the HSP60-p.Asp29Gly mutation causing a recessively inherited white matter disease called MitCHAP60 disease (OMIM #612233; Magen et al., 2008). Besides these two, a number of other variations have been described (Figure 1 and Table 1; Hansen et al., 2002, 2007; Bross et al., 2007). The Exome Aggregation Consortium webserver (ExAC; Cambridge, MA (URL: http://exac.broadinstitute.org) [June, 2016 accessed]) lists 95 missense variations for the protein product of the canonical *HSPD1* transcript. This website provides high quality exome sequencing data from more than 60,000 unrelated adults without severe pediatric disease. Of the 95 *HSPD1* missense variations 60 have only been seen in one single allele and only 9 have been observed in more than 10 of the ~120,000 alleles recorded (Table 1).

## Functional analyses of HSP60 variant proteins

Table 1 lists both the *HSPD1* missense variations described in the literature and those present in ≥10 alleles in the ExAC Consortium Website. The functional consequences for some of these variations have been investigated previously and the results are summarized in this review.

### *In vitro* analyses of disease-associated HSP60 variant proteins

Expression in *E. coli* had indicated that the two disease-associated variant proteins HSP60-p.Val98Ile and HSP60-p.Asp29Gly displayed similar stability as wild type HSP60 suggesting that the amino acid replacements caused functional impairment (Hansen et al., 2002; Magen et al., 2008). Indeed, the purified HSP60-p.Val98Ile protein assembled into native ring complexes in the same way and with similar efficiency as the wild type HSP60 protein but displayed a reduced ATPase hydrolysis rate and a severely decreased *in vitro* refolding activity with malate dehydrogenase as substrate (Bross et al., 2008). The ATPase function and refolding activity of the purified HSP60-p.Asp29Gly variant protein associated with MitCHAP60 disease was found decreased in a similar way (Parnas et al., 2009). This study also indicated impaired stability of HSP60-p.Asp29Gly oligomers causing their disassembly at low protein concentrations.

### Functional analysis *in vivo*

A sophisticated genetic complementation assay for analyzing the function of variants of the human HSP60/HSP10 chaperonin complex has been developed in the lab of Costa Georgopoulos (Richardson et al., 2001). This assay is based on knocking out the chromosomal *groESgroEL* operon and testing whether cell viability and growth properties can be maintained by providing the bacterial cells with a plasmid encoding the chaperonin gene variants to be investigated. Knock-out cells are not viable, but providing the cells with plasmids with cDNAs encoding the wild type human HSP60 and HSP10 genes maintains cell viability demonstrating that the human HSP60/HSP10 complex can functionally replace the bacterial GroEL/GroES complex (Richardson et al., 2001).

Seven human HSP60 missense variants have so-far been investigated using this functional assay (Table 1). These studies showed that the SPG13-associated mutant HSP60-p.Val98Ile was unable to functionally replace the bacterial chaperonin. However, cells expressing the HSP60-p.Asp29Gly variant displayed slow and temperature-sensitive growth. Cells with the HSP60-p.Gln461Glu variation found in a sporadic spastic paraplegia patient also displayed impaired growth. The four other HSP60 variants studied: p.Asn184Ser, p.Asp379Gly, p.Gly559Asp, and p.Gly563Ala, behaved like wild type HSP60 in the genetic complementation assay suggesting that they have no significant effect.

Bioinformatics prediction of the effects of the variations using the PolyPhen-2 tool (Adzhubei et al., 2010) predicts damaging effects for the HSP60-p.Val98Ile and HSP60-Gln461Glu variations, but benign for the HSP60-p.Asp29Gly for which a clear disease relationship has been established. PolyPhen-2 also predicts “damaging” for the two carboxy-terminal variations and four of the six missense variants found in more than 10 alleles in the ExAC dataset. Given the discrepancy between experimental and prediction results this prediction results must be taken with caution. Inspection of the position of the variations in the crystal structure of the HSP60/HSP10 complex (Nisemblat et al., 2015) shows that the variations with an established disease-association (HSP60-p.Asp29Gly and HSP60-p.Val98Ile) are localized in the core of the equatorial domain of the HSP60 protein, whereas most of the other variation sites are localized on the surface of HSP60 subunits (Figure 1A).

The SPG13-associated mutation HSP60-p.Val98Ile is dominantly inherited, i.e., the patient cells express both a wild type and a mutant allele and these two variant proteins are likely on equal terms incorporated into HSP60 ring structures resulting in heteromeric rings with stochastically distributed content of the two variants. To test whether incorporation of mutant HSP60-p.Val98Ile subunits together with wild type HSP60 subunits into HSP60 ring complexes would cause a dominant negative effect, the complementation assay was further engineered. *E. coli* cells with the deletion of the endogenous *groESgroEL* operon and containing a plasmid with an IPTG-inducible operons comprising HSP10 and wild type HSP60 were transformed with a second plasmid comprising an arabinose-inducible operon with the respective mutant HSP60 variant and HSP10 (Bross et al., 2008). There was no effect on growth in cells expressing both wild type HSP60 and HSP60-p.Val98Ile. As a control, expression of an artificially constructed ATPase-deficient HSP60 variant together with wild type HSP60 blocked growth. These experiments strongly indicated that the HSP60-p.Val98Ile mutation exerts no significant dominant negative effect on co-expressed wild type HSP60 protein.

## Clinical phenotypes of SPG13 and MitCHAP60 disease

Notwithstanding the rarity, studies of the very large index family that led to the discovery of the association of the HSP60-p.Val98Ile mutation with HSP has given a firm basis for the mutation/disease relationship (Hansen et al., 2002). SPG13 is dominantly inherited with a pure spastic paraplegia phenotype with high penetrance (Fontaine et al., 2000). Hereditary spastic paraplegia is a complex disease both genetically and clinically with mutations in more than 60 different genes established as causal and with all inheritance modes (Kumar et al., 2015; Tesson et al., 2015; Di Fabio et al., 2016). The characteristic spastic gait has given the acronym SPG and subsequent numerals distinguish the different genetic forms of HSP. Spastic gait and spasticity in the lower limbs are also observed as a side phenotype in many other neurological diseases. The disease is only classified as HSP if the paraplegia is the major clinical characteristic. Clinical analysis of the 10 MitChap60 patients homozygous for the HSP60-p.Asp29Gly mutation revealed also spastic paraplegia in all them (Magen et al., 2008). However, this “side-phenotype” was overshadowed by the much more severe presentation characterized by early-onset, profound cerebral involvement and lethality of these patients. A number of other SPG genes are also disease genes in other neurological diseases illustrating that maintaining the classical distinctions becomes more and more difficult (Tesson et al., 2015). Rather, the phenotype in a given patient depends on multiple factors, like the mutated gene, the nature of the mutation and its location in the protein, the zygosity of the mutation, and influences of modifier variants and the environment.

## Potential disease-association of other missense variations in HSP60

In spite of widely spread genetic screening, so far only one single additional spastic paraplegia patient heterozygous for another mutation in HSP60 (HSP60-p.Gln461Glu) has been reported (Hansen et al., 2007). The causative nature of this mutation is uncertain because two siblings carrying this mutation were asymptomatic. However, as the genetic complementation test showed a mild functional impairment (See Section Functional analysis *in vivo*), this variation may be disease-associated with reduced penetrance. The most frequently observed amino acid variation in HSP60, HSP60-p.Gly563Ala, has been found in homozygous form in one sporadic Danish spastic paraplegia patient (Svenstrup et al., 2009). Frequency analysis of this polymorphisms in Danish controls showed an allele frequency of 1.3% and similar allele frequencies were observed in all ethnic groups in the ExAC database. In addition, the number of homozygotes in the ExAC database is consistent with Hardy Weinberg distribution and the genetic complementation assay did not indicate impaired function of the HSP60-p.Gly563Ala protein. Taken together this suggests that this variation has no significant effect on function (Bross et al., 2007).

## Cellular and mouse models for HSP60 deficiency

Effects of expressing the SPG13- and MitCHap60-associated mutant proteins on mitochondrial morphology have been assessed in Cos-7 cells transfected with cDNAs encoding the disease-associated mutant proteins (Miyamoto et al., 2015, 2016). Cos-7 cells expressing either the mutant variants HSP60-p.Asp29Gly, HSP60-p.Val98Ile or HSP60-p.Gln461Glu displayed increased mitochondrial fission and decreased mitochondrial membrane potential whereas Cos-7 cells transfected with wild type HSP60 cDNA did not. This indicates that the studied mutant proteins interfere with the function of the endogenous wild type protein.

ShRNA-mediated knock-down of HSP60 in human HEK293 cells decreased the steady state levels of the mitochondrial medium-chain acyl-CoA dehydrogenase (Corydon et al., 2005), an enzyme whose subunits transiently interact with HSP60 before assembling into functional tetramers (Yokota et al., 1992; Saijo et al., 1994). Folding of ectopically expressed mitochondria-targeted green fluorescence protein (GFP) was decreased both in HEK293 cells in which HSP60 was knocked down and in HEK293 expressing a dominant negative ATPase-deficient HSP60 variant (Corydon et al., 2005; Bie et al., 2011). Furthermore, a series of assays of HSP60 knock-down in the mouse hypothalamic cell line N25/2 revealed decreases in mitochondrial respiration, levels of respiratory chain subunits, mitochondrial DNA levels and an increase in mitochondrial volume and mitochondrial superoxide (Kleinridders et al., 2013). These cellular models can be used for further studies elucidating the multiple effects of deficiency of the HSP60/HSP10 chaperonin complex.

Knock-out of both HSP60-encoding alleles in mice is not compatible with life. Such embryos died early during development (Christensen et al., 2010). However, mice which are heterozygous for one HSP60 knock-out allele, and which express half levels of HSP60 protein, developed normally and were borne in the expected Hardy-Weinberg frequency. More thorough long-term analysis of these mice revealed a late onset and slowly progressive deficit in motor functions recapitulating features of HSP SPG13 in humans (Magnoni et al., 2013). The phenotype was accompanied by morphological changes of mitochondria in spinal cord axons. Furthermore, decreased ATP synthesis was observed in mitochondria isolated from brain cortex and spinal cord. The respiratory chain defect could be narrowed down to impaired activity of respiratory chain complex III. Proteomic analysis of mitochondria from mutant mouse tissues consistently revealed decreased levels of the UQCRC1 subunit of complex III in these tissues. As UQCRC1 transcript levels were even increased and an effect on translation is improbable, this suggested that deficiency of the HSP60/HSP10 chaperonin complex resulted in impaired folding of the UQCRC1 protein entailing premature degradation of the UQCRC1 protein. Based on the same criteria and supported by direct interaction with HSP60, the manganese-dependent superoxide dismutase SOD2 was identified as another protein that is highly dependent on appropriate HSP60/HSP10 chaperone complex function (Magnoni et al., 2014).

Proteins like UQCRC1 or SOD2 thus appear to depend more than others proteins on folding assistance by the HSP60/HSP10 complex. For *E. coli* 85 proteins that display obligate dependence on folding assistance by the bacterial chaperonin complex have been characterized (Kerner et al., 2005). Identification of those proteins whose folding obligatorily requires the human HSP60/HSP0 complex is still lacking. Such knowledge would be very helpful to identify further mitochondrial functions affected by deficiencies of the HSP60/HSP10 complex.

## Perspectives

Different mutations in HSP60 or its partner protein HSP10 lead to distinct phenotypes of neurological disorders with a clear mitochondrial dysfunction pattern. These diseases are very rare as deleterious effects of mutations in these essential genes are not compatible with normal embryonal development (Christensen et al., 2010). The broader use of exome sequencing will likely reveal more cases also including *de novo* mutations in the *HSPD1* and *HSPE1* genes in sporadic diseases. Clinical geneticists should therefore be aware of this and it will be important to collect the knowledge of these rare cases to be able study genotype/phenotype relationships and to assess the disease-causing nature of variations.

Besides being affected by mutations, the activity and function of the HSP60/HSP10 complex can also be regulated by its expression levels. The regulation of the transcription levels of both proteins occurs via different elements in the bidirectional promoter. SP1 elements provide robust house-keeping levels of expression and on top of that heat-shock elements, mitochondrial unfolding protein response elements, and STAT3 elements further modulate expression adapting it to specific situations (Zhao et al., 2002; Hansen et al., 2003; Horibe and Hoogenraad, 2007; Kim and Lee, 2007; Kim et al., 2007; Kleinridders et al., 2013). One study also shows that Hsp60 mRNA is a direct target of miR-1 and miR-206 in cardiomyocytes (Shan et al., 2010).

Indeed, dysregulation of HSP60 expression in hypothalamus has been implicated with type 2 diabetes mellitus (Kleinridders et al., 2013) and changes in chaperonin expression and activity have been observed in several diseases such as cardiomyopathies, autoimmune disorders, and cancer (Cappello et al., 2014).

Finally, posttranslational modifications of the HSP60/HSP10 complex may regulate the activity of the complex. Like other molecular chaperones HSP60 is a highly modified protein with a long list of PTMs recorded in the UniProt database (Consortium, 2015): phosphorylation, acetylation, succinylation, malonylation, nitrosylation, sumoylation, ubiquitination, N-glycosylation, and O-GlcNAcylation. Acetylation of the co-chaperonin HSP10 has been indicated to affect activity of the chaperonin complex (Lu et al., 2014). One of the HSP60 missense variations shown in Table 1, Lysine-156, has been described before as modified by acetylation both in the human acute myeloid leukemia cell line MV4-11 (Choudhary et al., 2009) and in mouse liver HSP60 (Rardin et al., 2013). The p.Lys156Arg missense variation is classified as benign by the bioinformatics tools due to conservation of the positive charge, but elimination of this acetylation site may affect regulation of HSP60 function. Regulation of the activity of the HSP60/HSP10 complex at different levels may thus be a crucial hub for development of mitochondrial dysfunction, a hallmark in many disease processes.

## Author contributions

PF and PB have both contributed to writing the draft and producing the final version.

### Conflict of interest statement

The authors declare that the research was conducted in the absence of any commercial or financial relationships that could be construed as a potential conflict of interest.
